# Effectiveness and mediators of change of an online CBT intervention for students with adjustment disorder—study protocol for a randomized controlled trial

**DOI:** 10.1186/s13063-023-07744-9

**Published:** 2023-12-01

**Authors:** A. Juszczyk-Kalina, P. Holas, T. J. Farchione

**Affiliations:** 1 Department of Psychology, University of Warsaw, Warsaw, Poland; 2 Boston University, Boston, USA

**Keywords:** Adjustment disorder, Students, Internet psychological interventions, Cognitive behavioural psychotherapy, Daily diary study

## Abstract

**Background:**

Adjustment problems and disorders are highly prevalent among university students worldwide. These problems can cause significant interference in academic and social functioning and increase vulnerability to other mental health disorders. Unfortunately, only half of students in need receive psychological help. Furthermore, few studies have evaluated psychological interventions for adjustment disorders in students. New, more scalable forms of treatment for students with an adjustment disorder need to be developed, evaluated, and implemented. The study aims to determine the effectiveness of an online transdiagnostic cognitive behavioural intervention for students experiencing adjustment disorder and to assess mediators of change.

**Method/design:**

In this three-arm randomized controlled trial, we plan to recruit 214 Polish students diagnosed with an adjustment disorder. Participants who meet initial eligibility criteria will be randomly assigned to one of three 6-week conditions: (1) online cognitive behavioural therapy intervention based on an existing, empirically supported transdiagnostic protocol, the unified protocol; (2) online progressive muscle relaxation training as an active control group; or (3) waiting-list control group. Both interventions are asynchronous, interactive, and include minimal amount of therapist support. Assessments will consist of self-report questionnaires, daily diary measures, and neurocognitive tasks for evaluating cognitive functioning. These will be conducted at baseline, post-treatment, and 1-month follow-up. Daily diary measures will be taken during the first and last week of treatment (or waitlist period). Primary outcome measures will include adjustment disorder severity; secondary outcome measures will consist of other negative (psychopathology: depression, anxiety, and stress) and positive (life satisfaction) indexes of mental health as well as process measures (e.g. mindfulness, experiential avoidance, cognitive fusion).

**Discussion:**

To our knowledge, the current study is the first to evaluate the effectiveness of a psychological intervention for students with adjustment disorder. Therefore, it may have important practical implications for students with this disorder. It can potentially guide the development of a scalable, validated treatment option.

**Trial registration:**

Clinical Trials, NCT05768308, registered 14 March 2023, https://www.clinicaltrials.gov/ct2/show/NCT05768308

## Introduction

Students face many developmental challenges when transitioning to a university setting, such as leaving the family home, becoming independent, and adjusting study habits for the university setting [1]. These challenges make them vulnerable to mental health problems, especially adjustment disorders. Research has shown that the prevalence of mental health problems in this group is higher than in the general population [2, 3].

Despite a clear need for mental health treatment, students rarely seek out and use in-person psychological services [4, 5]. Potential reasons for this include privacy concerns, low emotional openness, and financial issues [6]. Self-help online therapy programs can address many of these problems [7, 8]. They require fewer clinical resources and provide the possibility of help without fear of losing privacy. A survey conducted in 2020 in the USA showed that 71% of students would like to use this approach to treatment [9]. This proportion has continued to increase over time; in 2010, the percentage was only 58% [10]. Such interventions are currently used in the USA and a few countries worldwide; despite their promising effects, they are not widespread [11]. Unfortunately, no such intervention is available for students in Poland.

Adjustment disorder (AjD) is a disruption of adjustment following a stressful situation [12]. ICD-11 [13] lists revised criteria for disorders that require the presence of a psychosocial stressor and a stress reaction in the form of symptoms stemming from absorbing the stressor or its consequences, as well as clear disturbances in adaptation to the situation.

Several variables associated with AjD can be understood as transdiagnostic processes underlying theory of behavioural change in acceptance and commitment therapy (ACT). This includes psychological flexibility and inflexibility processes. Psychological inflexibility is understood as a failure to change one’s behaviour due to entanglement with inner experiences (e.g. thoughts and feelings), despite the detrimental or ineffective nature of the behaviour [14]. Inflexibility processes associated with AjD include experiential avoidance and cognitive fusion [15, 16]. The combination of these two processes has been found to play a significant role in causing and maintaining psychological distress [17, 18]. Also, negative repetitive thinking, which can be understood as a maladaptive experiential avoidance strategy, predicts psychological adjustment [19, 20].

On the other hand, psychological flexibility processes associated with AjD are mindfulness and self-compassion [21, 22]. They have been proposed to attenuate the effects of experiential avoidance and cognitive fusion on well-being (e.g. [23, 24]). Evaluating whether these variables are mechanisms of changes in psychological interventions aimed at AjD is essential.

Unfortunately, there is little research on AjD as a clinical diagnosis among students, and mental health among students is a relatively neglected area [1]. Previous studies have focused mainly on problems with academic adjustment, specifically, adjusting to new challenges connected to life at university. Results of these studies indicate that academic adjustment problems are highly prevalent (around 50%) and negatively impact students’ functioning, including worse grades, high university dropout rates, and poor mental health [25–29]. They are also associated with suicidal ideation [30, 31]. There is also a significant association between academic adjustment problems and the diagnosis of AjD [29].

Since the prevalence of academic adjustment problems is so high among this group and only half of students in need receive psychological help [32], it is important to develop and examine the effectiveness of an alternative—an online, self-help form of psychological assistance for students with AjD. It is also important to investigate the mechanisms of its effectiveness. Within the present literature on protecting students’ mental health, online therapy has been presented as an effective and potentially scalable solution [33]. To our knowledge, there has only been one study to date; it was focused on an online psychological intervention aimed at students with academic adjustment difficulties. It was based on an online meditation app available for commercial use. The study results suggest that individuals who used the app more frequently experienced more significant improvements in academic adjustment. Unfortunately, the study was limited by low app usage [34].

Internet interventions contain classic psychotherapy principles adapted to an online form. They can be fully automated—without psychotherapist support (unguided)—or allow therapist support [35]. They have been implemented worldwide for 20 years, but this area is still in its infancy in Poland [36], where there is a lack of both adapted e-interventions and research on their effectiveness [36]. Global data show that such programs can effectively treat mental disorders and be used in prevention efforts among student populations [37, 38].

Several studies have been published assessing the effectiveness of online therapeutic interventions aimed at people with AjD. All were based on CBT principles. Their promising results show small to medium effect-size improvements in experimental groups [39–42]. Unfortunately, these studies struggled with high dropout rates (50–80%) during the intervention. The percentage of people not helped by the interventions was also significant [43]. These studies relied on self-reported questionnaire methodologies, where it was impossible to determine any changes obtained in participants’ daily lives (at work, at school, or in family life). We intend to employ a more robust methodology and assess outcomes important for life in a university environment—such as cognitive functioning and academic performance. We will also study daily diary entries to increase the validity of results and obtain a more nuanced understanding of treatment effects.

Researchers have identified several factors that may improve the effectiveness of online psychotherapeutic interventions, such as interactive content, shorter intervals between sessions, and targeting the intervention to a specific type of stressor or a specific population [43, 44]. Several of these suggested changes will be incorporated into the intervention used in the present trial.

AjD is a disease with various clinical forms, with a predominance of depression, anxiety, or anger; therefore, transdiagnostic cognitive behavioural therapy (CBT) raises hope for increasing the effectiveness and clinical utility of treatment for this disorder. A study conducted among college students in 2013 confirmed that transdiagnostic CBT is an effective form of treatment for AjD [45]. The unified protocol (UP)—which will be used in the current study—is a structured, manualized transdiagnostic intervention for emotional disorders. The UP aims to address temperamental characteristics, particularly neuroticism and resulting emotion dysregulation, which are argued to be a common factor underlying all emotional disorders [46].

### Objectives

The main objective of the planned research is to evaluate the effectiveness of an online UP intervention among students experiencing AjD.

More specific objectives are as follows: (1) evaluate the effectiveness of the intervention relative to waiting-list and active control groups according to self-reported measures; (2) investigate the effectiveness of the intervention in the daily lives of participants; (3) assess the effectiveness of the intervention in the cognitive functioning of students; (4) conduct an examination of predictors and mediators of treatment response; (5) compare the impact of both treatment approaches, with different putative mechanisms of action, on outcome measures; and finally (6) evaluate the maintenance of AjD alleviation at 1-month follow-up.

## Methods/design

### Study design

This study is a parallel group randomized controlled trial (RCT). The participants will be allocated to one of three 6-week conditions: (1) online cognitive behavioural therapy, based on the unified protocol (iCBT-UP); (2) online progressive muscle relaxation training (iPMR, active control group); and (3) waiting-list control group (WLC). The last group will receive access to their chosen intervention after the waiting period (6 weeks). To help WLC participants maintain motivation, weekly emails will be sent to remind them of the study and to assess their mood. They will be instructed to contact the research team in instances of any crisis. At that point, they will be administrated by our team psychotherapist or psychiatrist and referred to an adequate service. Assessments will consist of questionnaires, a cognitive task and a study of daily diaries. All assessments will be administered through an online data collection platform delivered by organisers (Supporty.pl), occurring at (1) screening, (2) baseline (prior to randomization), (3) at the end of the 6-week online program (or waitlist period), and (4) 1 month after finishing treatment (for participants in the active intervention conditions). Daily diary studies will be carried out for entries in the first and again in the last week of the interventions. Cognitive task will be administered at baseline, at the end of 6 weeks of online intervention, and 1 month after finishing treatment. The study will follow SPIRIT guidelines (Standard Protocol Items: Recommendations for Interventional Trials) [47].

**Fig. 1:**
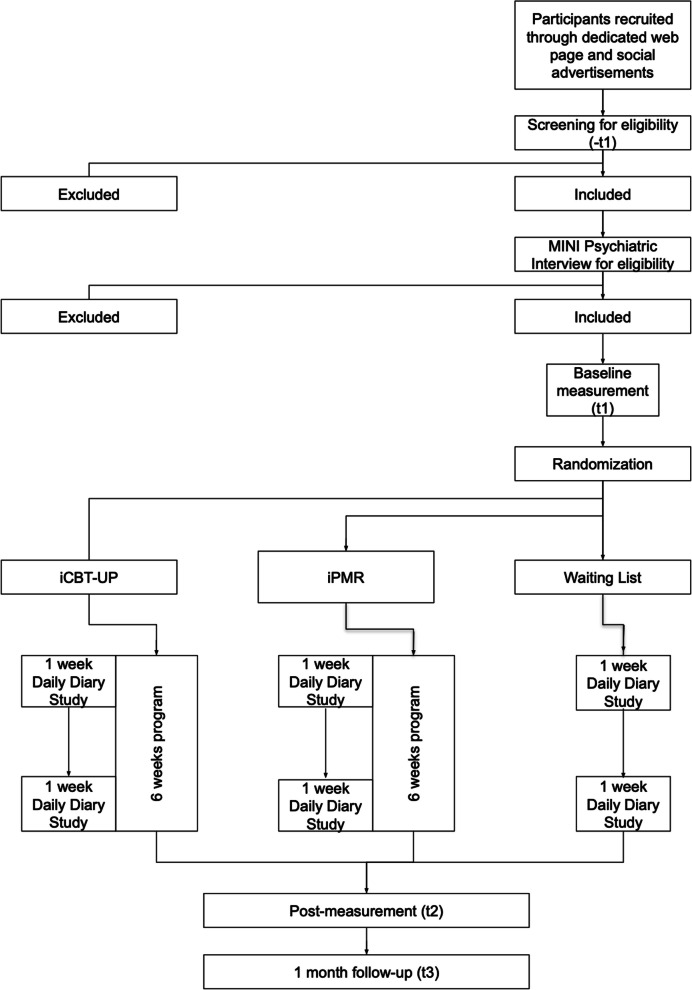
Design of the trial

Figure 1 shows the trial design. The study protocol and informed consent have been approved by the Ethics in Research Committee of the Faculty of Psychology, University of Warsaw.

### Participant recruitment and inclusion and exclusion criteria

Participants will be recruited using announcements in popular social media and university newsletters. The study will be advertised to Polish students from different universities around the country. Interested participants will provide informed consent and then complete screening questionnaires. Those meeting preliminary eligibility criteria will then be assessed using the Mini-International Neuropsychiatric Interview (MINI) to ensure they meet Diagnostic and Statistical Manual of Mental Disorders, Fifth Edition (DSM-5) [48] diagnostic criteria for an AjD as a primary diagnosis and to establish comorbidities. The diagnostic interview will be administered by a trained psychologist or psychotherapist by video conference and then entered to the database.

The inclusion criteria consist of having student status, being 18 years old or older, having access to the internet and using a computer or smartphone, and meeting the diagnostic criteria of AjD in the IADQ questionnaire and meeting diagnostic criteria for AjD in the MINI.

The exclusion criteria consist primarily of conditions that require prioritization for immediate or simultaneous treatment: specifically, an increased risk of suicidality intent or ideation, bipolar and psychotic disorder, and/ or alcohol/substance use disorder. Individuals will also be excluded if they are receiving another psychological treatment at the start of the study or have no time to participate in the program. Pharmacological treatment during the intervention period will be allowed, provided that the type and dose of medication are stable for 6 weeks before study involvement.

Individuals not meeting the inclusion criteria will be referred to adequate services.

### Randomization and blinding

Subjects who meet the inclusion criteria will be randomized to one of two active groups or the waiting-list group using a 2:2:1 allocation ratio. The participants will be randomly allocated using a computerized block randomization, using block sizes of 6. Random allocation will be performed using an algorithm developed by a computer scientist and executed independently of the research team by an independent program [49]. The main researcher will then reveal the concealed allocation and inform participants of their placement into one of three groups. Randomization will be stratified based on the score of the initial AjD questionnaire assessment (the IADQ; see description below). The principal investigator responsible for determining study eligibility will be blinded to the randomization sequence. The researchers and psychotherapists leading the study and contacting participants cannot be blinded to allocation for practical reasons; however, those conducting the MINI evaluation will be blind to the condition allocation during the assessment.

### Risk management

Internet interventions used in the present study were not designed to provide crisis support for persons experiencing suicidal crises. We will exclude individuals with suicidal ideations and refer them to adequate services. We will also monitor participant mood using daily diary measures during the first and last week of the intervention and weekly throughout the intervention, by contacting participants through a chat function to assess any risk. In instances of crisis, we will organise a meeting with our team of psychotherapists and provide crisis support or refer the individual to an adequate service (psychotherapy, psychiatrist, or hospital).

### Interventions

The interventions are self-help digital programs that will be provided to participants through an online delivery platform. The participants will gain access to their randomly selected intervention via a self-generated password and email address. Access to programs will be provided for 8 weeks; the programs will be 6-week long. All conditions will include an anonymous chat function whereby participants can share opinions and ask the research team for help. The participants will also receive minimal therapeutic support. To this end, they will receive weekly emails introducing new program modules and containing motivating prompts. Additionally, once a week, they will receive a message on chat. The aim of this communication is to assess the mood of participants and provide brief feedback on their work to enhance their motivation.

#### Internet CBT-UP intervention

The cognitive-behavioural intervention developed for this study is based on transdiagnostic CBT principles of the UP [50]. Three systematic reviews, with two meta-analyses, have been conducted to evaluate the efficacy of the UP. These reviews reveal that the UP significantly improves anxious and depressive symptoms with moderate to large effect sizes [51, 52]. Our iCBT-UP is a modular intervention consisting of six components teaching different skills for responding to strong negative emotions and emotion regulation difficulties and associated anxiety, depression, and related disorders. Several modifications were made to the original UP in developing the iCBT-UP. These include shortening the intervention program from 8 to 6 modules (see Table 1 for information). This procedure was implemented because of high dropout rates in longer online interventions. The introduction (module 1) and summing-up part (module 8) in the iCBT-UP intervention are not considered treatment modules; they have been shortened to focus exclusively on main goals and are presented as a “pop-up” section upon first entering the intervention site and after completing all modules.

**Table 1:** Overview of iCBT-UP intervention

Number	Name	Description
Introduction		Participants learn about objectives of the program and can set their own goals
Module 1	Understanding Your Emotions	Participants learn about the nature and function of emotions. Users learn that all emotions can be broken down into 3 components: thoughts, physical sensations and behaviours, and that each interacts with the others
Module 2	Mindful Emotion Awareness	This module introduces mindful emotion awareness as a skill to use in response to emotion. The two components of this module are present-focused awareness and non-judgemental awareness. Participants can practice these components by audio guided exercises
Module 3	Flexible Thinking	Participants learn about the relationship between their emotions and thoughts. They have a chance to acknowledge their thoughts in different situations, learn about thinking traps and practice flexible thinking
Module 4	Emotional Behaviours	Participants learn about the relationship between their behaviours and emotions. They come to know what we understand by emotional behaviours and think about how they express themselves in their own lives. In the exercises, they learn how to change emotional behaviours
Module 5	Facing Physical Sensations	Participants learn about the effect of physical sensations. They induce physical sensations and learn how to tolerate and accept them
Module 6	Emotion Exposures	At the end, participants set a hierarchy of emotionally difficult situations and conduct emotion exposures
Summary		A brief summary of the content of all modules; relapse prevention

The iCBT-UP used in this study is an interactive intervention with text and audio materials, videos, quizzes, pictures, and gap-filling exercises. Each module consists of an introductory part—with a short video and text and an examination of previous knowledge—followed by a didactic component with activities to train new skills and personal stories where the participants become acquainted with the experiences of two students who are followed throughout the program.

We will recommend completing one module per week, but participants will have the option to complete the program at their own pace.

#### Internet progressive muscle relaxation training (iPMR)

Training in the comparison condition is based on PMR principles [53]. Studies have shown that relaxation reduces stress, anxiety, and depression among university students [54]. One of the most often used researched relaxation techniques is PMR [55]. PMR has been proven to reduce college students’ anxiety and stress and boost happiness [56, 57]. In the present study, we want to evaluate whether changes obtained in iCBT-UP will be significantly higher, than those obtained in an active, well-researched comparator. The PMR training aims to teach how to quickly achieve a state of relaxation. It is also a modular intervention. It consists of five modules (the first module lasts 2 weeks). See Table 2. Participants are asked to practise techniques from each module twice a day. The intervention is also interactive and contains videos introducing new topics to participants and audio-guided relaxations.

**Table 2:** Overview of iPMR intervention

Number	Name	Description
Module 1	Progressive Muscle Relaxation of 16 Group of Muscles	Participants learn how to tighten and relax 16 groups of muscles. This module is recommended to last for 2 weeks
Module 2	7 Muscle Group Relaxation	This is a shortened version of PMR. Participants learn how to tighten and relax 7 groups of muscles (first arm, second arm, head, neck, torso, first leg, second leg)
Module 3	4 Muscle Group Relaxation	This is a shortened version of PMR. Participants learn how to tighten and relax 4 groups of muscles (arms, head, torso, legs)
Module 4	Release Only Condition	Module in which participants are taught to obtain a state of relaxation without tightening muscles
Module 5	Your Own Experience	In this module, participants are engaged to train relaxation on their own—without audio recordings. They can do so in different situations in their daily lives and in different body positions

#### Waiting List Control (WLC)

We included the WLC condition to evaluate, if changes obtained in the iCBT-UP group can be attributed to the intervention, rather than simply the passage of time. This group will receive access to active intervention (iPMR or iMBCT) after the 6-week waiting period and completion of the post-treatment assessment.

### Measures

A summary of all measures and time points is presented in Table 3.

**Table 3:** A description (SPIRIT diagram) of enrolment, intervention, and assessment

	**Study period**						
	**Enrolment**	**Allocation**	**Baseline**	**During the 6 weeks interventions**		**Post assessment**	**Follow-up**
**Timepoint**	***-t***_***1***_	**0**	***t***_***1***_	***Daily Diaries week (1–7 day of intervention)***	***Daily Diaries week (36–42 day of intervention)***	***t2*** ***(1 day after the intervention)***	***t3*** ***(30 days after the intervention)***
**Enrolment:**							
**Eligibility screen**	X						
**Informed consent**	X						
**Allocation**		X					
**Interventions:**							
***iCBT-UP***				X	X		
***iPMRT***				X	X		
***Waiting list***				X	X		
**Assessments:**							
***MINI***	X						
***IADQ***	X					X	X
***PHQ-9***	X					X	X
***GAD-7***	X					X	X
***SWLS***			X			X	X
***AAS***			X			X	X
***PSS-10***			X			X	X
***FFMQ-15***			X			X	X
***AAQ-II***			X			X	X
***SCS-SF***			X			X	X
***CFQ***			X			X	X
***PTQ***			X			X	X
***WEMWBS***			X			X	X
***Daily diaries***				X	X		
***Cognitive task***			X			X	X
***Adverse events***						X	

#### Diagnostic measure

The Mini-International Neuropsychiatric Interview (MINI) [58] is a short diagnostic interview based on DSM-5 criteria, which focuses on the existence of current psychiatric disorders. It consists of separate modules to diagnose specific disorders.

#### Questionnaires

##### Primary outcome measures

The International Adjustment Disorder Questionnaire (IADQ) [59] is a 19-item questionnaire used to assess AjD related to ICD-11 criteria [13]. The IADQ consists of nine item stressor list, three items that assess preoccupation with the stressor, three items that consider symptoms of failure to adapt, three items that assess functional impairment, and one item that assess duration of symptoms. The primary endpoint for this outcome is immediately post intervention. Then, we will assess it after a one-month follow-up to evaluate the stability of the obtained changes. We will compare post-test scores between three conditions as well as change from baseline measurement.

##### Secondary outcome measures

The Scale of Experienced Stress (PSS-10) [60] is a scale made to measure stress levels and consists of 10 items. Higher scores indicate higher perceived stress.

The Academic Adjustment Scale (AAS) [61] is a nine-item scale that measures students’ academic adjustment levels. It has three subscales (three items on each): academic lifestyle, academic achievement, and academic motivation. It is widely used among permanent residents and sojourner students.

The Satisfaction With Life Scale (SWLS) [62] is a five-item self-report global measure of life satisfaction.

The Patient Health Questionnaire-9 (PHQ-9*)* [63] is a method used to assess levels of depressive symptoms. It consists of nine statements derived from the Diagnostic and Statistical Manual of Mental Disorders, Fourth Edition (DSM-IV) criteria for depressive disorder and an additional statement regarding the severity of existing symptoms in daily life.

The General Anxiety Disorder-7 (GAD-7) [64] is a measure used to assess levels of anxiety symptoms in generalized anxiety disorder (GAD) defined by the DSM-IV. The scale contains seven items related to GAD characteristics (feeling anxious, worrying too much, having difficulties relaxing, etc.).

The Five Facet Mindfulness Questionnaire (FFMQ-15) [65] is a 15-item self-report measure of mindfulness. It contains the same five facets as the longer form: observing, describing, acting with awareness, non-judging of inner experience, and non-reactivity to inner experience. It includes three items for each facet.

The Acceptance and Action Questionnaire (AAQ-II) [66] is the most widely used measure of psychological inflexibility and experiential avoidance. It consists of seven items rated on an 8-point Likert-like scale. Higher scores indicated greater psychological inflexibility. Developed initially as a direct measure of psychological inflexibility, later studies have highlighted how the AAQ-II could be more suited as an indicator of experiential avoidance [67].

The Self-Compassion Scale—Short Form (SCS-SF) [68] is a 12-item questionnaire designed to measure the capacity for self-compassion. The SCS-SF has two subscales: self-disparagement and self-care—six items on each. High levels of total score are characterized by high self-care and low self-disparagement.

The Cognitive Fusion Questionnaire (CFQ) [69] is a brief, unidimensional seven-item self-report measure of cognitive fusion.

The Perseverative Thinking Questionnaire (PTQ) [70] is used to measure repetitive negative thinking. It consists of 15 items, each associated with one of the process characteristics of perseverative thinking: (1a) intrusive (e.g. “Thoughts come to my mind without me wanting them to”), (1b) difficult to disengage from (e.g. “I can’t stop dwelling on them”), (2) unproductive (e.g. “I keep asking myself questions without finding an answer”), and (3) capturing mental capacity (e.g. “My thoughts prevent me from focusing on other things”).

The Warwick-Edinburgh Mental Well-Being Scale (WEMWBS) [71] is a measure of mental well-being. It consists of 14 items rated on a 5-point Likert scale. The higher the global score, the higher the level of mental well-being.

#### Daily diary assessment

The daily diary assessment will contain two parts.

The first part will be described as a daily diary of life events. In this part, participants are asked to recall all important events that happened that day. Using a 7-point Likert scale, participants will rate each event in terms of stressfulness and presence (how mindful they were). The second part contains questions evaluating daily well-being and depressogenic adjustment. All daily measures used in our study have been previously used in research [72, 73]. Each measure is based on a corresponding trait measure of the same construct, with items selected for appropriateness for daily administration and reworded. All daily items include the word “today.”

The following variables will be measured:

Depressogenic adjustment will be measured by three items based on Beck’s [74] triad.

Procrastination—measured by three items from the Pure Procrastination Scale [75].

Daily negative thinking—worrying and rumination. Daily worrying will be measured with three items taken from Meyer et al.’s scale [76]. Daily rumination will be measured with three items based on Trapnell and Campbell’s scale [77].

Emotional regulation—cognitive restructuring and emotional suppression. These variables will be measured using one item (each scale) from the Emotional Regulation Questionnaire (ERQ [78]).

Self-compassion—measured by three items from the SCS [68].

Daily affect—to be measured based on the circumplex model [79]. This model contains a combination of positive–negative, active–deactive emotions that produce four measures. Positive active affect is measured with ratings for feelings described as happy, proud, and excited/enthusiastic, and positive deactive affect is measured with ratings for those described as calm, satisfied, and relaxed. Negative active affect is measured with ratings for feelings described as upset, stressed, and angry, and negative deactive affect is measured with ratings for those described as sad, bored, and disappointed. Participants will be describing how they feel using a 7-point Likert scale.

#### Cognitive task

To measure the cognitive functioning of students, we will use an online version of the Stroop task [80] (color–word), adapted from Mead et al. [81]. It assesses cognitive flexibility and attention span. We will use the color–word condition and present on the screen names of colors printed with the ink of a different color (blue, red, green, yellow). The subject must name the color in which the stimuli are presented and disregard their verbal content by pressing the correct button. The color of the ink and the presented word are never the same. The Stroop test is a widely used measure in research on students’ mental health and cognitive functioning [82–84].

#### Adverse events

Post-treatment, adverse events will be assessed using a self-reported question, “Did you experience any negative effects during treatment? If yes, please give us further details” [85]. Then, the answers will be coded using unwanted effects checklist [86].

### Record retention and confidentiality

Participants who proceed to the trial assessment will receive a trial code. Organizers will record and store all data collected in the study in an electronic database on a secure server build. All databases will be anonymized—all identifiers will be irreversibly removed, and subjects will no longer be identifiable. All principal investigators will have access to a secured database.

### Withdrawal from the trial

Participants may voluntarily withdraw from the trial for any reason at any moment.

### Sample size calculation

The sample size was determined in relation to the between-group effect size and calculated using G*Power software. Previous research on an online UP intervention and online interventions for AjD has revealed a between-group effect size at the level of *d* = 0.5 and a within-group effect size at the level of *d* = 0.63 [42, 87]. Assuming an alpha 0.05 with a power of 0.80 and post-treatment between-group effect size of *d* = 0.5 (iCBT-UP versus control), 190 participants in the three groups will be sufficient to detect assumed differences. However, because the literature reveals dropout rates from online CBT interventions, with minimal guidance, of approximately 30% [88], a total sample of 247 participants will be recruited.

### Data analysis

All data will be analysed using the Statistical Package for the Social Sciences (SPSS) version v21.0 (IBM Corporation, Armonk, NY, USA). All randomized participants will be analysed, including those who stop receiving treatment on an intention-to-treat (ITT). The multiple imputation method will be used for missing data when appropriate. We also plan to perform sensitivity analysis to test the robustness against departures from the assumption of missing data being at random following the technique explained in Crameri et al. [89]. Hierarchical linear modelling will be used to assess our primary and secondary outcomes, daily diary results, and cognitive functioning. The most detailed level of analysis will contain results from daily diary measurements. The next level will be based on differences between each measurement. The last level in the model will include differences between each of the three groups. Hayes’ Process Macro will be used to assess mediation effects [90]. In this approach, effects are assessed using bias-corrected bootstrap confidence intervals. These effects are considered significant if the upper and lower bounds of the bias-corrected 95% confidence intervals do not contain zero. Mediation is assessed through the indirect effect of the independent variable on the dependent variable, mediated by an intermediate variable. For each statistically significant result, effect size will be calculated. The number of times each participant uses the program will be used to measure adherence. The results of this study will be submitted for publication in a peer-reviewed journal.

### Governance and oversight of the trial

We will establish a trial management group (TMG) that will oversee the whole process of the research—recruitment of participants, assessments, ethical issues, adverse events, and data management. This group will meet once a week during the active phase of the research and in every case as the need arises. The group will consist of the principal investigator, the supervisor and co-principal investigator, and the trial platform manager. Due to the latest recommendations, adverse events will be investigated twofold. First is using our questionnaire outcome measures—to determine the number of patients who deteriorated and those who did not respond to the treatment. Second is using a self-report measure to detect adverse events administered post-treatment (described above) [85]. In case of any changes to the protocol, the TMG will communicate them to the investigators and participants and record them in the study diary.

## Discussion

To our knowledge, there is no published study evaluating the effectiveness of an online psychological intervention for students experiencing AjD. Our study is meant to fill this gap. The study aims to examine the effectiveness of an internet psychological intervention for students experiencing AjD. Further objectives are to explore mechanisms for obtained changes. We expect that the online UP intervention will be an effective form of treatment, and we expect that obtained changes will be significantly greater than those from the two control conditions. We also predict that experiential avoidance, mindfulness skills, self-compassion, and cognitive fusion will mediate this effect.

The study has several strengths: first, it was designed as an RCT with two control conditions. A meta-analysis of online psychological interventions for students in 2019 revealed that only 23% of evaluated studies used an active control condition [11]. Therefore, we will recruit not only a passive control group but also an active one—also an online intervention, namely PMR training. Our study is also aimed at evaluating its effectiveness using several different measurement approaches, in addition to self-report questionnaires, including a daily diary study and a cognitive task. We state that the outcomes of the intervention should be representative of the university environment rather than only depression, anxiety, or AjD, which tend to span all life domains. These issues have largely remained unaddressed in studies examining the effectiveness of online psychological interventions for students. For example, in the abovementioned meta-analysis, only 9 out of 48 studies used a student functioning outcome [11]. Our study is also intended to examine the psychological mechanisms of the obtained changes. Although assessing the efficacy of the intervention is our primary goal, understanding how the intervention works (mechanisms of change) is no less important. It is essential for several practical reasons [91]. First, identifying variables responsible for changes obtained in the intervention can help in future iterations of the intervention; they can be redefined to focus on these mechanisms to maximize effectiveness. Secondly, interventions can be shortened to the pieces contributing to the outcome. Thirdly, it can help distinguish between specific and non-specific treatment effects. Unfortunately, there is little research on, and in Poland, there are few studies of, the mediators of change in therapeutic internet programs (Wardeszkiewicz J, Holas P: Internet-delivered modified Mindfulness-Based Cognitive Therapy (iMBCT) intervention for perceived stress and emotional distress: a pilot RCT study, under review).

The field of psychological internet interventions in Poland is still in its infancy. To our knowledge, there is only one such study in Poland conducted on a population of people experiencing AjD. The study evaluated the efficacy of online mindfulness-based cognitive therapy (eMBCT) [92]. Its results were promising and showed a significant reduction of AjD symptoms in the experimental group compared with the control condition. We have been unable to find a study involving an internet-based psychological intervention in Poland aimed at college students. However, recent research outcomes have shown that 64% of students consider access to free psychological help for students in Poland insufficient; 67% of students think that forms of psychological care at universities are insufficient [93]. Clearly, there is an urgent need to implement and research new methods of psychological help for students. Our study addresses this need and may have important practical implications.

Nevertheless, these strengths will probably be associated with limitations.

First, previous research findings have shown high dropout rates in studies of internet psychological interventions [94]. Low adherence might be especially problematic for the present study, as the large number of assessments combining traditional questionnaires with a daily diary may lead to a relatively high burden for participants. However, we plan to implement financial rewards for participants and other ways of increasing motivation and commitment among participants, including motivational email and communication via chat, that hopefully will promote their adherence [95]. In addition, to deal with the problem of high dropout rates, we plan to gather a relatively large number of participants. Furthermore, we have limited the daily diary period to 7 days at the beginning and the end of the study and included only short assessment scales that are essential to the study.

### Trial status

This is the first version of the protocol, dated 2 February 2023. Recruitment will commence on 1 November 2023, with an estimated completion date of 1 March 2024. If we do not gather enough participants, we will continue the ongoing recruitment until we reach the estimated group sample size.

## Data Availability

When results will be published in a peer-reviewed journal, fully anonymized data will be made available on request to other researchers.

## Abbreviations

AAS: Academic adjustment scale
AAQ-II: Acceptance and Action Questionnaire
ACT: Acceptance and Commitment Therapy
AjD: Adjustment disorder
CBT: Cognitive behavioural therapy
CFQ: Cognitive Fusion Questionnaire
DSM-IV: Diagnostic and Statistical Manual of Mental Disorders, Fourth Edition
DSM V: Diagnostic and Statistical Manual of Mental Disorders, Fifth Edition
ERQ: Emotion Regulation Questionnaire
FFMQ-15: Five Facet Mindfulness Questionnaire
GAD-7: General Anxiety Disorder-7
IADQ: International Adjustment Disorder Questionnaire
ICBT-UP: Internet Cognitive Behavioural Therapy—Unified Protocol
ICD-11: International Classification of Diseases, Eleventh edition
iPMR: Internet Progressive Muscle Relaxation
MBCT: Mindfulness-based cognitive therapy
MINI: Mini-International Neuropsychiatric Interview
PHQ-9: The Patient Health Questionnaire-9
PTQ: Perseverative Thinking Questionnaire
PSS-10: Scale of experienced stress
RCT: Randomised controlled trial
SCS-SF: Self Compassion Scale—Short Form
SPIRIT: Standard Protocol Items: Recommendations for Interventional Trial
SPSS: Statistical Package for the Social Sciences
SWLS: Satisfaction with life scale
TMG: Trial management group
UP: Unified protocol
WEMWBS: Warwick-Edinburgh Mental Well-being Scale
WLC: Waiting List Control
